# Genomic surveillance of SARS-CoV-2 reveals highest severity and mortality of delta over other variants: evidence from Cameroon

**DOI:** 10.1038/s41598-023-48773-3

**Published:** 2023-12-08

**Authors:** Joseph Fokam, Rene Ghislain Essomba, Richard Njouom, Marie-Claire A. Okomo, Sara Eyangoh, Celestin Godwe, Bryan Tegomoh, John O. Otshudiema, Julius Nwobegahay, Lucy Ndip, Blaise Akenji, Desire Takou, Mohamed M. M. Moctar, Cleophas Kahtita Mbah, Valantine Ngum Ndze, Martin Maidadi-Foudi, Charles Kouanfack, Sandrine Tonmeu, Dorine Ngono, John Nkengasong, Nicaise Ndembi, Anne-Cecile Z. K. Bissek, Christian Mouangue, Chanceline B. Ndongo, Emilienne Epée, Nadia Mandeng, Sandrine Kamso Belinga, Ahidjo Ayouba, Nicolas Fernandez, Marcel Tongo, Vittorio Colizzi, Gregory-Edie Halle-Ekane, Carlo-Federico Perno, Alexis Ndjolo, Clement B. Ndongmo, Judith Shang, Linda Esso, Oliviera de-Tulio, Moussa Moise Diagne, Yap Boum, Georges A. E. Mballa, Louis R. Njock, Genomic Surveillance Study Group, Serge Alain Sadeuh Mba, Paul-Alain Tagnoukam Ngoupou, Moumbeket Yifomnjou Henri, Bertrand Eyoum, Grace Beloumou, Guy Pascal Ngaba, Christiane Medi, Lydie Nyatte, Melissa Sanders, Marie Amougou, Loko Bille, Kizito Atehambe Buyohnwenda, Claudine Ngomtcho, Abas Mouliom, Fai Karl Gwei Njuwa, Gisele Nke Ateba, Alex Nka, Laura Dimite, Adama N. Dir, Carole Eboumbou

**Affiliations:** 1 National Public Health Emergencies Operations Coordination Centre (NPHEOCC), Ministry of Public Health, Yaoundé, Cameroon; 2 COVID-19 Genomic Surveillance Platform (PSG), Ministry of Public Health, Yaoundé, Cameroon; 3 Chantal BIYA International Reference Centre for Research on HIV/AIDS Prevention and Management (CIRCB), Yaoundé, Cameroon; 4 Faculty of Health Sciences (FHS), University of Buea, Buea, Cameroon; 5 National Public Health Laboratory (NPHL), Ministry of Public Health, Yaoundé, Cameroon; 6 Faculty of Medicine and Biomedical Sciences (FMBS), University of Yaounde I, Yaounde, Cameroon; 7 Centre Pasteur du Cameroun (CPC), Yaoundé, Cameroon; 8 Centre de Recherche en Maladies Emergentes et Re-emergentes (CREMER), Yaounde, Cameroon; 9 School of Public Health, University of California, Berkeley, Berkeley, CA USA; 10 World Health Organization (WHO), Cameroon Country Office, Yaounde, Cameroon; 11 Centre de Recherche Pour la Santé des Armées (CRESAR), Ministry of Defence, Yaoundé, Cameroon; 12 USAID’s Infectious Diseases Detection and Surveillance, Yaounde, Cameroon; 13 African Society for Laboratory Medicine (ASLM), Yaounde, Cameroon; 14 Africa Centres for Disease Control and Prevention (Africa CDC), Addis-Ababa, Ethiopia; 15 Division for Operational Health Research (DROS), Ministry of Public Health, Yaoundé, Cameroon; 16 Department of Disease, Epidemic and Pandemic Control (DLMEP), Ministry of Public Health, Yaounde, Cameroon; 17 Faculty of Medicine and Pharmaceutical Sciences (FMPS), University of Douala, Douala, Cameroon; 18 Faculty of Health Sciences (FHS), University of Bamenda, Bamenda, Cameroon; 19 Institut de Recherche Pour le Developpement (IRD), Montpellier, France; 20 Chair of UNESCO Biotechnology, University of Rome Tor Vergata, Rome, Italy; 21 Bambino Gesu Pediatric Hospital, Rome, Italy; 22 US Centres for Disease Control and Prevention (CDC), Cameroon Country Office, Yaounde, Cameroon; 23 University of KwaZulu-Natal and Stellenbosch University, Stellenbosch, South Africa; 24 Institut Pasteur de Dakar, Dakar, Senegal; 25 Epicentre, Medecins Sans Frontières (MSF), Yaounde, Cameroon; 26 General Secretariat, Ministry of Public Health, Yaounde, Cameroon

**Keywords:** Biotechnology, Computational biology and bioinformatics, Microbiology, Molecular biology, Medical research, Molecular medicine

## Abstract

While the SARS-CoV-2 dynamic has been described globally, there is a lack of data from Sub-Saharan Africa. We herein report the dynamics of SARS-CoV-2 lineages from March 2020 to March 2022 in Cameroon. Of the 760 whole-genome sequences successfully generated by the national genomic surveillance network, 74% were viral sub-lineages of origin and non-variants of concern, 15% Delta, 6% Omicron, 3% Alpha and 2% Beta variants. The pandemic was driven by SARS-CoV-2 lineages of origin in wave 1 (16 weeks, 2.3% CFR), the Alpha and Beta variants in wave 2 (21 weeks, 1.6% CFR), Delta variants in wave 3 (11 weeks, 2.0% CFR), and omicron variants in wave 4 (8 weeks, 0.73% CFR), with a declining trend over time (p = 0.01208). Even though SARS-CoV-2 heterogeneity did not seemingly contribute to the breadth of transmission, the viral lineages of origin and especially the Delta variants appeared as drivers of COVID-19 severity in Cameroon.

## Introduction

The global report on coronavirus disease 2019 (COVID-19) revealed 770,563,467 confirmed cases (including 20,917,453 active cases) and 6,957,216 deaths (i.e. 0.9% case fatality rate [CFR]) in 224 affected countries as of September 7, 2023^1^. In Africa, 54 countries have been affected by the pandemic, with 12,837,874 confirmed cases and 258,830 deaths (i.e. 2.1% CFR) across the continent. In Cameroon, there were 125,165 confirmed cases (including 34 active cases) and 1,974 deaths (1.6% CFR) across all health districts following the COVID-19 situation report of August 27, 2023^2,3^.

COVID-19 pandemic has been characterised by several epidemiological waves and the emergence of new severe acute respiratory syndrome coronavirus 2 (SARS-CoV-2) variants from the ancestral strain from Wuhan, China^4^. Variants are classified according to their level of significance as variants of high consequence (VHC), variants of concern (VOC), variants of interest (VOI) or variants under monitoring (VUM)^5,6^. While VHCs have not yet been reported, several VOCs have been identified as the driving force of viral circulation and dispersal, with higher burdens reported in Northern Europe, Central America, and sub-Saharan Africa^7,8^.

Regarding the dynamics and spread of VOCs over time, the Alpha (B1.1.7), Beta (B1.351), and Gamma (P1) variants were among the first emerging viral clades, with a foremost circulation of Alpha; Delta variant (B1.617) then emerged predominantly (faster, fitter and more transmissible compared to previous variants), and finally the emergence of Omicron variant (B1.1.529) showed the highest viral fitness over previously known VOCs^9^. With these rapid changes in SARS-CoV-2 patterns, it is of paramount importance to establish a strategy for variant surveillance in order timely mitigate their potential impacts^10^. Of the 10,293,748 whole-genome sequences available by April 22, 2022, in the Global Initiative on Sharing All Influenza Data (GISAID), Omicron variant represents approximately half, followed proportionally by the Alpha, eta, Delta, and Gamma variants^11^.

In Cameroon, the first COVID-19 case was detected on March 6, 2020^12^, and the country has experienced five different waves with varying outbreak magnitudes, durations, number of confirmed cases and hospitalisations, number of severe or critical cases, number of deaths, and CFR^12^. The hypothetical variability in the clinical features and epidemiological trends warrants investigating on possible implications of SARS-CoV-2 variants on the dynamics of the pandemic^13^. Such genomic investigation would contribute in designing context-specific public health measures as part of the pandemic response strategies. Of note, SARS-CoV-2 genomic surveillance can shed light on the origins of viral lineages (imported or emerging locally), the transmission dynamics and phylogeography of these viruses, and their potential clinical relevance (disease severity) and public health implications (transmissibility) at the national level^13^. In this frame, we sought to ascertain the introduction and dynamics of SARS-CoV-2 lineages and their effects on transmission and disease severity following the various epidemiological waves in the Cameroonian context.

## Results

Based on whole-genome sequences of SARS-CoV-2 from Cameroon deposited in GISAID between August 2021 and March 2022, a total of 760 individual samples from Cameroonian residents were enrolled in the present study.

The mean age of the study population was 36 (min–max: 2–86) years and 45.0% were within the age range 26–45. Regarding gender distribution, 50.9% were male and 49.1% female.

### Distribution of the study population with whole-genome sequences by region of residence

Samples were from 9/10 regions of Cameroon (Table 1) and classified into three categories according to sampling/proportions:

- three regions with high proportions of samples (≥ 10%): Centre, Littoral, and Southwest;
- two regions with moderate proportions of samples (between 5 and 10%): East and West;
- four regions with low proportions of samples (< 5%): North, South, Adamawa, and Far-North.

**Table 1:** Distribution of the study samples according to region of origin.

Region	Number	Percentage
Adamawa	17	2.2%
Centre	373	49.1%
East	45	5.9%
Far-North	11	1.4%
Littoral	142	18.7%
North	27	3.6%
West	44	5.8%
South	25	3.3%
South-West	76	10.0%
Total	760	100.0%

This geographical distribution indicates that only 30% of the regions in Cameroon had an acceptable coverage for SARS-CoV-2 genomic surveillance nationwide.

### Diversity of SARS-CoV-2 lineage from whole-genome sequencing

**Figure 1:**
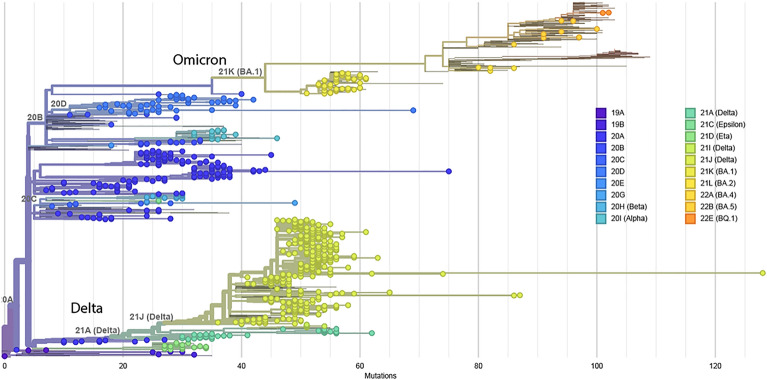
Phylogenetic tree of SARS-COV-2 lineages when using whole-genome sequences. Figure is composed of 760 individual genome sequences. The collection dates range from 2020-03-06 to 2022-02-02; Data were collected in one country and territory; all sequences in this dataset were rooted with the hCoV-19/Wuhan/WIV04/2019 (WIV04), the official reference sequence employed by GISAID (EPI_ISL_402124), https://gisaid.org/WIV04.

Phylogenetic analysis of the 760 whole-genome sequences revealed that the greater proportion of SARS-CoV-2 variants circulating in Cameroon belonged to the viral sub-lineages of the ancestral strain from Wuhan (74%), 15% Delta, 6% Omicron, 3% Alpha and 2% Beta variant (Fig. 1). The observed distribution reflects the high number of samples processed for genomic surveillance at the early phase of the pandemic (see Supplementary materials, SDC1 and SDC2).

### Dynamics of SARS-CoV-2 lineages over time

**Figure 2:**
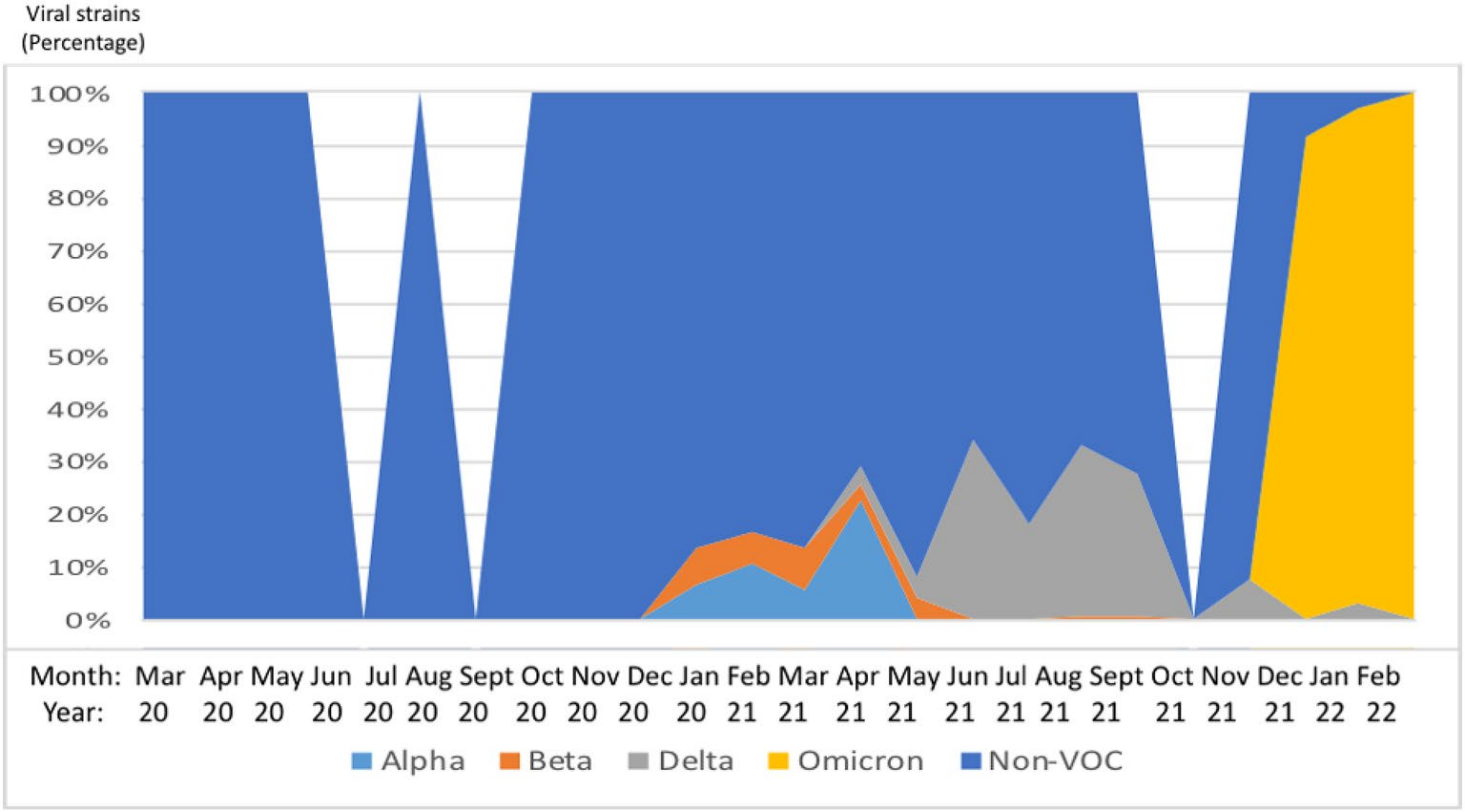
SARS-COV-2 lineage dynamics per month in Cameroon. X-axis represents month-year of sampling; Y-axis represents the percentage of SARS-CoV-2 strains; colours correspond to each viral strain.

Throughout the study reporting period, the patterns of SARS-CoV-2 evolved over time. From March 2020 to November 2020, the introduction of the cases of SARS-CoV-2 occurred, solely with viruses of the lineage of origin. In December 2020, first cases of Alpha and Beta variants were identified and remained in circulation till May 2021 (for Alpha) and June 2021 (for Beta). First cases of the Delta variant were identified in March 2021, with the number of cases increasing substantially until October 2021, followed by a slight upsurge between November 2021 and January 2022. Finally, first cases of Omicron emerged by September 2021 overtaking the Delta variant to reach 100% circulation in February 2022 (Fig. 2).

### Variations in major SARS-CoV-2 lineages according to epidemiological waves

**Figure 3:**
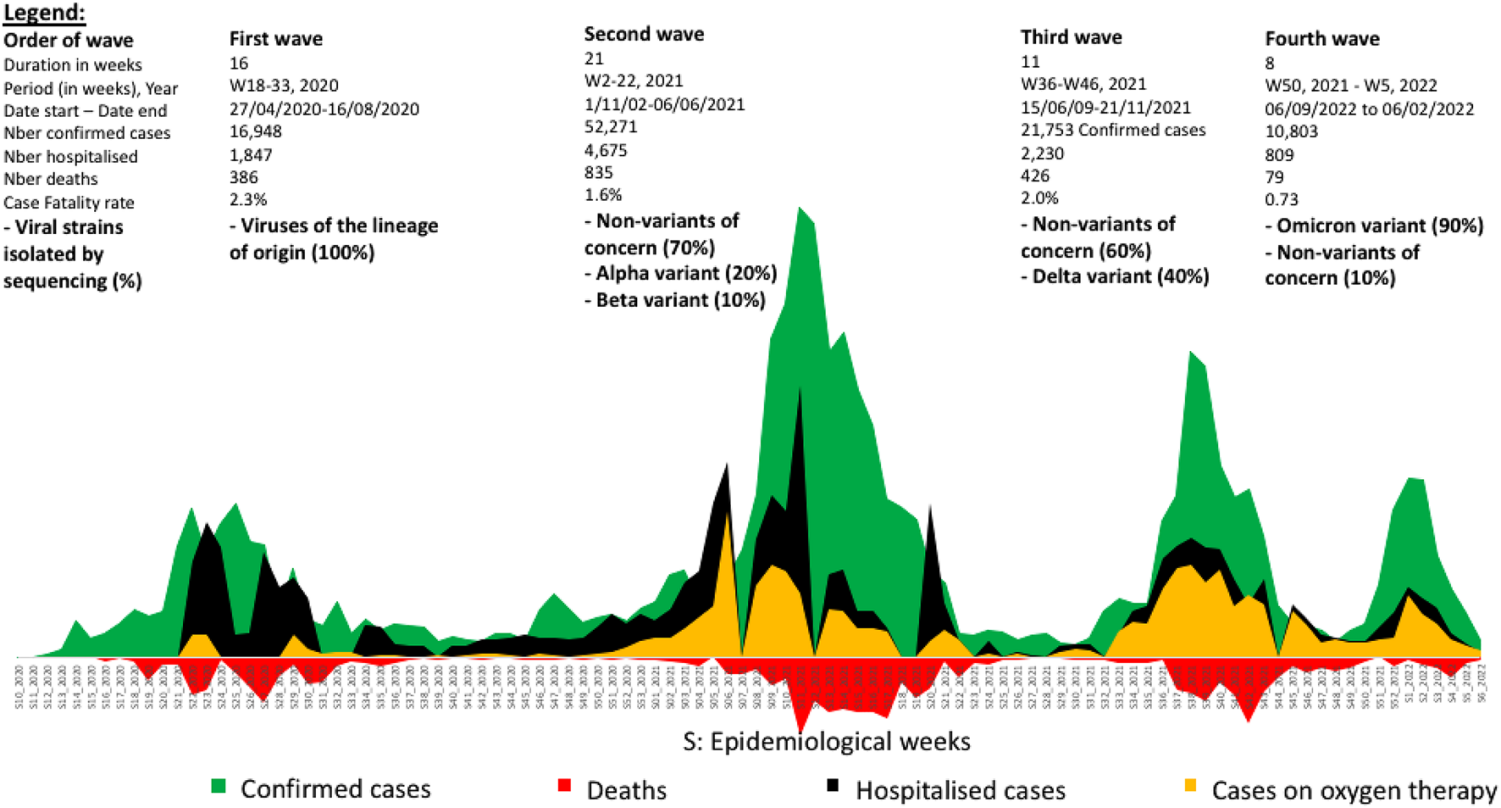
SARS-CoV-2 lineage dynamics per wave in Cameroon. The x-axis shows the epidemiological week and year (i.e. S10_2020 means week10_year2020); colours correspond to specific clinical conditions (confirmed cases, hospitalised, deaths, oxygen therapy). The following data are provided by wave: the order of wave; the duration (in weeks); the period-year of the wave; date start–date end; number confirmed cases per wave; number of hospitalised cases per wave; number of deaths per wave; case fatality rate per wave; viral strains isolated per wave.

Figure 3 provides the trends in duration, number of confirmed cases, number of deaths, and the CFR from one wave to another.

i. *During the first wave*, the epidemic was driven by SARS-CoV-2 lineages of origin/non-VOC; the outbreak duration was moderate (16 weeks); the number of confirmed cases was moderate (16,948); the number of hospitalised cases was high (1847); the number of deaths was moderate (386); and the CFR was high (2.3%).
ii. *During the second wave,* the epidemic was driven by the co-introduction of Alpha and Beta alongside SARS-CoV-2 lineages of origin/non-VOC; the outbreak duration was long (21 weeks); the number of confirmed cases was high (52,271); the number of hospitalised cases was high (4675); the number of deaths was high (835); and the CRF was moderate (1.6%).
iii. *During the third wave*, the epidemic was driven by Delta alongside SARS-CoV-2 lineages of origin/non-VOC; the outbreak duration was moderate (11 weeks); the number of confirmed cases was high (21,753); the number of hospitalised cases was high (2230); the number of deaths was moderate (426); and the CFR was high (2.0%).
iv. *During the fourth wave*, the epidemic was mainly driven by Omicron; the outbreak duration was short (8 weeks), the number of confirmed cases was moderate (10,803), the number of hospitalised cases was low (809), the number of deaths was low (79); and the CFR was low (0.73%).

### Correlation between the wave duration and number of cases according to variant dynamics

**Figure 4:**
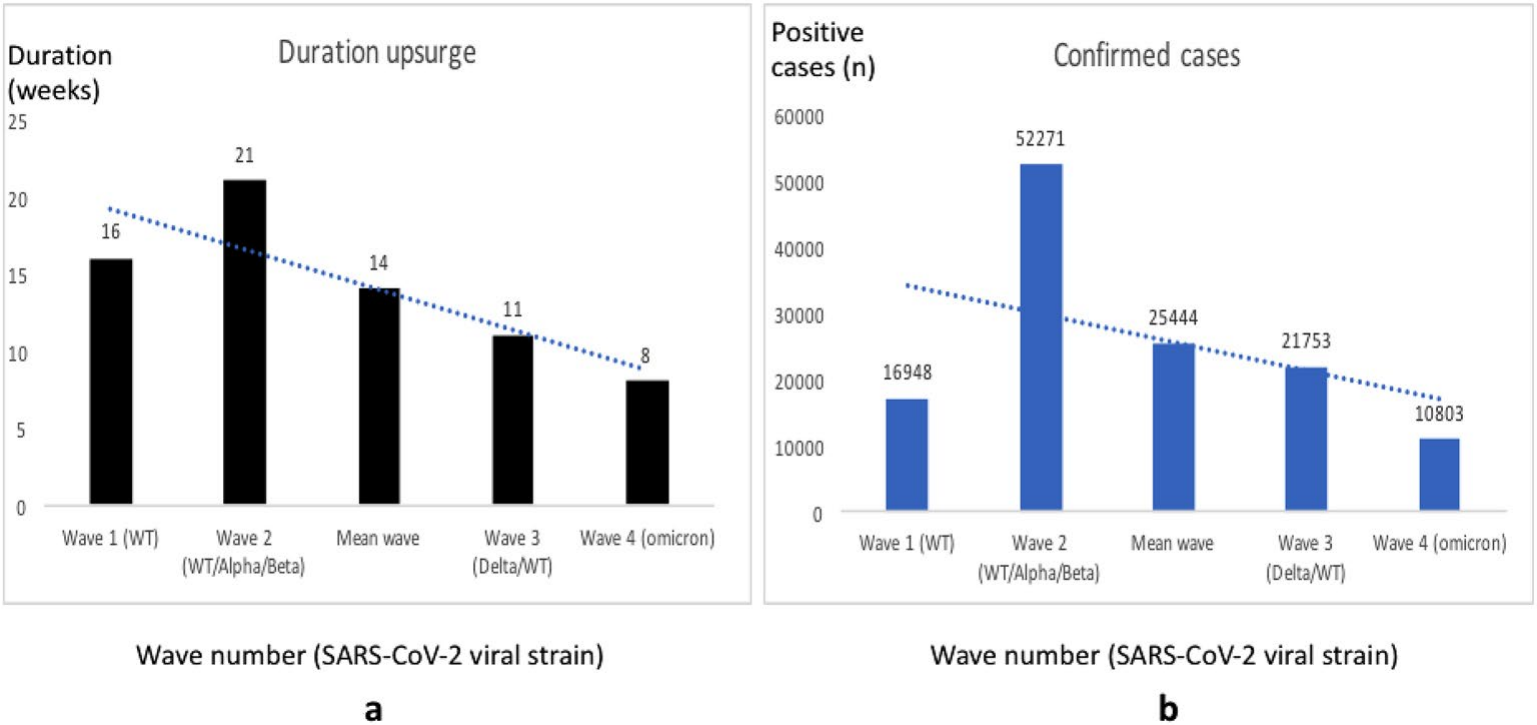
Correlation analysis between the wave length and the number of confirmed cases following variant dynamics. (**a**) Duration of outbreak by wave; (**b**) Number of confirmed cases by wave, *WT* wild type.

Figure 4 presents the trend in the duration of each wave (Fig. 4a) and the number of cases per wave (Fig. 4b), alongside the detection of major circulating VOC for each wave.

From wave 1 to wave 4, there was an overall declining trend in the wave duration (mean duration: 14 weeks) as well as the number of confirmed cases per wave (mean value: 25,444). These trends showed a significant positive correlation between the wave duration and the number of confirmed cases (z score = − 2.50672; p = 0.01208), indicating that the dynamics of SARS-CoV-2 variants were not the primary drivers of the number of cases observed per wave. Hence, viral transmission was mainly driven by outbreak duration.

### Correlation between the number of hospitalisations and CFR according to variant dynamics

**Figure 5:**
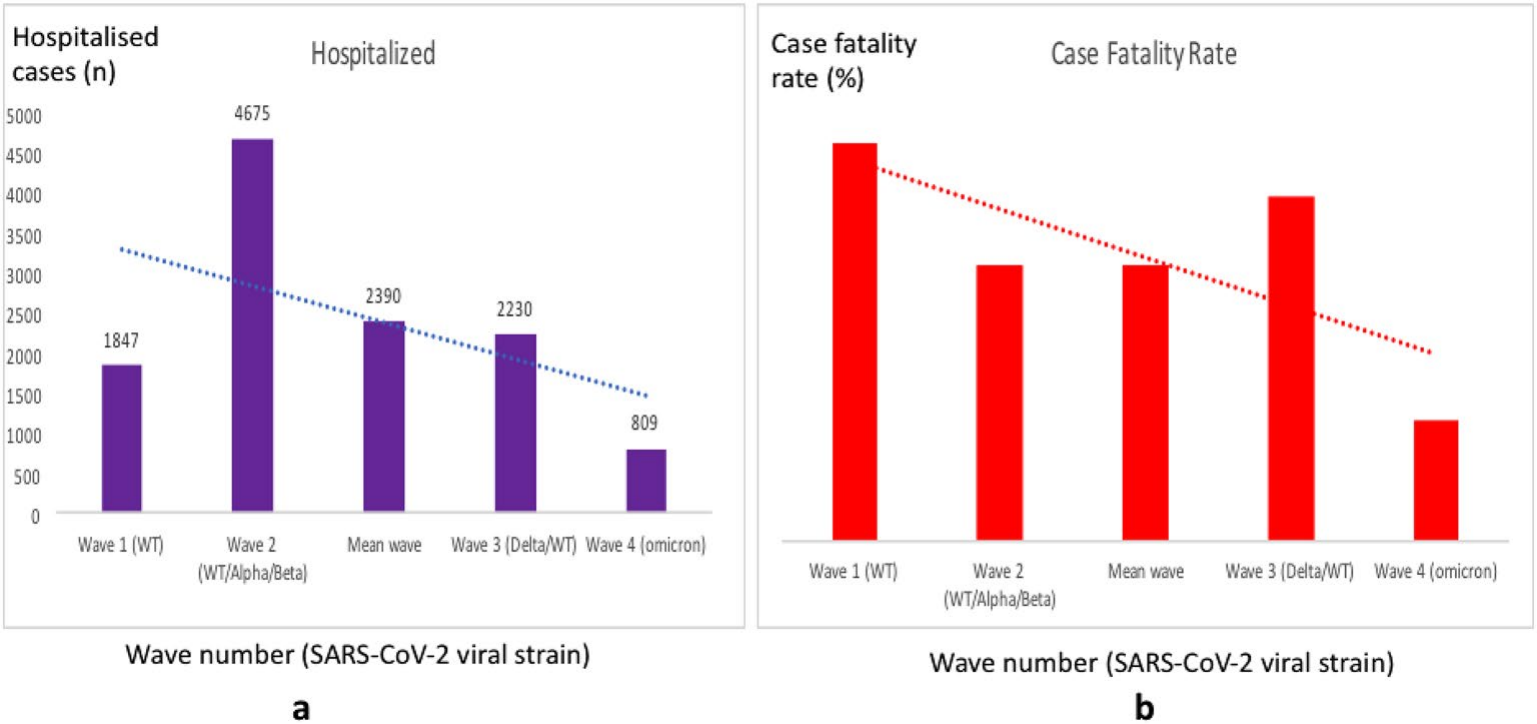
Correlation analysis between hospitalisation and CFR following variant dynamics. (**a**) Number of hospitalisations by wave; (**b**) Case fatality rate by wave, *WT* wild type.

From wave 1 to wave 4, there was an overall declining trend in the number of hospitalisations (mean: 2390 cases) and the CFR (mean value: 1.66) per wave (Fig. 5a and b, respectively). Despite the significant correlation (z-score = 2.50672; p = 0.01208), there was a discrepancy between the low number of cases and the high CFRs in Wave 1 and Wave 3. This suggests that the original viral lineage and the Delta variant contributed to the severity of COVID-19 in the Cameroonian context.

## Discussion

The present reveals the power of genomic surveillance for SARS-COV-2 in understanding the the dynamics in the epidemiology of COVID-19 at national level^13,16^. In settings with limited access to sequencing^17,18^, collaborative efforts enabled sampling and sequencing of SARS-CoV-2 through the genomic surveillance platform in place^19–21^, with international partnerships^13,16,22^. Thus, this initiative could be viable for any genomic surveillance of any pathogen of pandemic or epidemic potential (Ebola, Zika, Mpox viruses, cholera, antimicrobial resistant strains, etc.) as supported by Africa CDC^3^ and other agencies^18,23^.

In this study, the mean age of the population was 36 (26–45) years. This represents the most active population often involved in travels, social or occupational activities that increase exposure to SARS-CoV-2, as previously reported in similar settings^24–26^. However, this observation is different in the Western world most likely due to greater adherence to barrier measures^27^. The sex ratio showed a similar distribution in SARS-CoV-2 cases, indicating similar risk of infection/exposure at population-level^28^.

Sampling was from 9/10 of the national regions, suggesting wide near-national coverage (only North-West region excluded). However, only 30% of regions achieved a desirable sampling for genomic surveillance (at least 10% of sequence data). This is in line with coverage of molecular testing mostly found in major townships^25,29^. Thus, genomic data from difficult-to-reach settings are less covered^30^.

According to available sequence data, the viral sub-lineages of origin and non-VOCs represent the majority (74%) as compared to VOCs, reflecting efforts in genomic surveillance during the early phase of the pandemic even at the global level^31–33^. Interestingly, transmission dynamics confirms the first cases as viral sub-lineages of origin; followed between December 2020 and April 2021 by the co-introduction of alpha and beta as first VOCs, favoured by population migrations for end-of-year holidays from the Western world where these variants were already prevalent^31,34^. First cases of delta were identified in March 2021 with a peak in October 2021. The increase in cases with delta would be favoured by the variant affinity for the ACE2 receptor due to mutations^35^, leading to enhanced viral attachment, high viral load and prolonged duration of infection^36^. First cases of omicron were found in September 2021 and predominate as from December 2021 to reach 100% by end of February 2022. Regarding the evolutionary trends of variants per wave, the first wave (driven by lineages of origin) has a medium length in duration, a moderate number of cases and deaths, but a high CFR which could be attributed to limited experience/logistics of the health system to respond to an unknown disease^37^; not neglecting the effect of stigma and fear on late clinic attendance during that early phase^38,39^. The second wave was driven by the co-introduction of alpha and beta variants alongside other sub-lineages and non-VOCs, and was characterised by the longest outbreak duration of about five months, a high number of cases and hospitalisations, but a moderate CRF. The decreased CFR might be due to timely clinic attendance and gradual experience in case management^39,40^. Moreover, as compared to the high circulation of sub-lineages of origin and non-VOCs, alpha and beta variants had limited effects on transmission and disease severity, which prone their disappearance^41,42^. In contrast, the third wave (driven by the Delta variant) had a moderate duration of about three months but an increased/high number of confirmed cases (over 20,000), hospitalisations (over 1000) and high CFR (2%). Thus, in a relatively short timeframe, delta variant considerably increased both the transmission rate and the disease severity^41,42^. The fourth wave (driven solely by omicron) had a very short duration of about two months and a reduced/moderate number of cases (10,803) and low number of hospitalisations/deaths and CFR (0.73%), which underscores the contribution of omicron on viral transmission but without severity^43,44^. The overall decline in outbreak duration and number of cases across waves highlights the fact that the extent of viral transmission/spread was mainly driven by the duration of the outbreak. However, the low number of cases and high CFRs during wave 1 and wave 3 signifies that the original viral lineage and delta variant contributed to COVID-19 severity in the Cameroonian context, alongside other multifaceted determinants (naïve populations at the early stage of the pandemic, etc.)^25^. Thus, among VOCs, delta was a substantial driver of COVID-19 severity and death, likely attributed to loss in antibody affinity due to viral antigenic mutations in the receptor-binding domain^45^.

The main limitation of our study is the lack of proportionally representative samples across all regions and across the four waves. This suggests a reduced generalizability of the findings, especially in terms of overall prevailing viral lineages. Nonetheless, these findings, generated with high-quality and full-length sequences validated by a public repository (GISAID) and interpreted using a robust phylogenetic pipeline, provide evidence with major public health implications to prepare for future pandemics^15^.

In a nutshell, our genomic surveillance with full-length sequences reveals four VOCs (alpha, beta, delta, and omicron) in Cameroon across four different epidemiological waves. SARS-CoV-2 infection in Cameroon has been driven by the viral lineages of origin in wave 1, the co-introduction of alpha and beta variants in wave 2, delta variant in wave 3 and omicron variant in wave 4, with an overall declining trend in the wave duration, confirmed cases, hospitalisations and CFR over time. While viral transmission was not dependent on viral clades, SARS-CoV-2 viral sub-lineage of origin (at the early phase) and Delta variant appeared to be the drivers of COVID-19 severity in Cameroon.

## Methods

A laboratory-based survey was conducted within the framework of the national Public Health Emergencies Operations Centre (PHEOC) for COVID-19 in Cameroon, from March 1, 2020 to March 30, 2022, through an assessment of the evolutionary patterns of SARS-CoV-2 lineages across the four COVID-19 waves in the country.

### Specimen collection and referral for SARS-CoV-2 genomic surveillance

The identification, packaging, storage, and transportation of positive COVID-19 nasopharyngeal samples from the testing sites to the reference laboratories were performed by staff trained in field epidemiology. For every sample positive on antigen rapid diagnostic test (RDT), a swab was collected on viral transport medium (VTM) and transported using a triple packaging with a cold chain (refrigerated cooler) from the testing site to the PCR reference laboratory for molecular testing. For collection sites far from a PCR reference laboratory, samples were stored at − 20 °C and transported within 2–7 days to the nearest PCR reference laboratory.

### Nucleic acid extraction, amplification and detection of SARS-CoV-2

At the PCR reference laboratory, viral RNA was extracted from 140 µL nasopharyngeal swab using the QIAamp Viral RNA Mini Kit (Qiagen Inc, Valencia, CA, USA) as per manufacturer’s instructions. Amplification was performed using the DaAn gene detection kit for 2019-nCoV (https://en.daangene.com/uploads/file/detection-kit-for-2019-novel-coronavirus-2019-ncov-rna-pcr-fluorescence-probing.pdf). The protocol used probes targeting the open reading frame (ORF1ab) gene and the nucleocapsid (N) protein gene, with a lower limit of detection of 500 copies/mL and an amplification reaction of 45 cycles. Briefly, 03 µL of enzyme (solution B) and 05 µL of SARS-CoV-2 RNA were added into 17 µL of master-mix (solution A). The total (master mix and biological sample) was then placed into a thermocycler for reverse transcription (at 50 °C, 15 min); Taq pol activation (95 °C, 15 min); and finally amplification during 45 cycles (94 °C, 15 s and 55 °C, 45 s). RT-PCR results were interpreted as the presence of viral RNA for cycle threshold (CT) value ≤ 37 (i.e. PCR-positive) as per national guidelines.

The eligibility criteria for sequencing were as follows: a PCR-positive sample, a cycle threshold (CT) value < 30 for manual RT-PCR or equivalent, and a minimum volume of 200 μL of swab and/or 30 μL of viral RNA. Moreover, wherever necessary, eligible samples were stored at − 20 °C for a maximum of 30 days and shipped under a stable cold chain to the sequencing reference laboratory, along with a standard electronic metadata file.

### Whole-genome sequencing of SARS-CoV-2

Sequencing was performed using the Illumina protocol for whole genome. Briefly, libraries were generated using the amplicons generated; indexed paired-end libraries were prepared using the Nextera DNA Flex Library Prep Kits (Illumina) as per the manufacturer’s instructions. Each tagged amplicon was and barcoded with a unique barcode using the Nextera CD Indexes. Libraries were purified and normalized to 4 nM prior to pooling, and the pool was denatured using 0.2 N sodium acetate and then diluted to a final concentration of 8 pM. The library was spiked with 1% PhiX Control v3, and the libraries were sequenced using a 500-cycle v2 Reagent Kit as per the manufacturer’s instructions (Illumina, San Diego, CA, USA). Fastq files produced from Illumina MiSeq were assembled using Genome Detective (https://www.genomedetective.com/) and the coronavirus typing tool, and were visualized for quality using FastQC. Following cleaning of sequences, short reads are sorted and placed into groups and metagenomic de novo assembly was performed. Each group of sequence was then identified; Blastx and Blastn are used to search for candidate reference sequences against the NCBI RefSeq virus database. The results for all detected contigs are combined by the Advanced Genome Aligner and scored using by Genome Detective at the amino acid and nucleotide level. The five best scoring references for each config are then used for the alignment. Data on full-length sequencing were consecutively entered into the GISAID platform, under the following sequence accession numbers (from “hCoV-19/Cameroon/Yaounde-20V-3870/2020” to “hCoV-19/Cameroon/ECO284/2021”). These data were downloaded, and the molecular phylogeny of the SARS-CoV-2 sequences was performed using Nexstrain (see Supplementary Digital contents—SDC1)^15^. The fasta sequences a GenBank repository under the following accession number GenBank OQ520884-OQ521579. The phylogenetic analysis, the Nextstrain pipeline (https://github.com/nextstrain/ncov) was used to generate the build. Sequencing data from the rest of the world were included for phylogenetic context based on genomic proximity and even sampling over time, however for the Cameroon build, we zoomed in on the country-specific sequences. To prepare data for the Cameroon-specific Nextstrain analyses we included the sequence and metadata files. The customized workflow is available at the following link (https://nextstrain.org/groups/cameroon-genomics/Cameroon-ncov-build?c=clade_membership&f_country=Cameroon). To visualize the results, we employed Auspice to work with the output .json file generated in the Nextstrain environment.

A graphical representation of the dynamics of different SARS-CoV-2 variants identified during the outbreak was created using Excel version 2004. The wave durations according to detected VoCs were graphically displayed, along with the related epidemiological information. This provided a global picture of the evolution of SARS-CoV-2 variants during the first to the fourth waves of the pandemic in Cameroon for possible predictions toward the control of future outbreaks. Based on our local experience, the interpretation of severity by wave was performed, as shown in Table 2.

**Table 2:** Grading assessment of epidemiological and clinical conditions per wave.

	Low	Moderate/medium	High/long
Duration of the wave (weeks)	< 10	10–20	20
Number of confirmed cases	< 10,000	10,000–20,000	> 20,000
Number of hospitalised cases	< 500	500–1000	> 1000
Number of reported deaths	< 100	100–500	> 500
Case fatality rate (CFR)	< 1.0	1.0–2.0	> 2.0

A wave was considered to be of short, medium, or long duration if it lasted for < 10, 10–20, or > 20 weeks, respectively. The number of confirmed cases was defined as low, moderate, or high if it was < 10,000, 10,000–20,000, > 20,000, respectively. The number of hospitalised cases was classified as low, moderate, or high if < 500, 500–1000, or > 1000, respectively. The number of deaths was interpreted as low, moderate, or high if < 100, 100–500, or > 500, respectively, and the CFR was categorised as low, moderate, or high if < 1, 1–2, or > 2, respectively.

The Mann–Whitney U test was used to calculate the correlation between the duration of each outbreak and the number of confirmed cases and between hospitalised cases and CFR, with a p value < 0.05 and a Z-score ≥ 2 considered statistically significant.

### Ethical considerations

The present study was performed in accordance with the Declaration of Helsinki. Briefly, ethical clearance was obtained from the National Ethics Committee for research on human health (reference N°2022/01/1430/CE/CNERSH/SP/SP; N°2020/05/1224/CE/CNERSH/SP/SP), and administrative authorisation was provided by the Ministry of Public Health (N°368/NS/MINSANTE/SG/CCOUSP/CSO). Ethical clearance was also obtained from the Centres for Disease Control and Prevention. Each participant provided their informed consent and filled the case reporting form. Confidentiality was ensured by using fully anonymised data from a secured public database repository (GISAID) that was populated with metadata provided by the study team.

### Supplementary Information

Supplementary Information 1.

Supplementary Information 2.

Supplementary Information 3.

## Data Availability

Sequences were submitted to NCBI GenBank repository under the following accession number GenBank OQ520884-OQ521579. GISAID Identifier of the sequence dataset: EPI_SET_230214oa 10.55876/gis8.230214oa. All genome sequences and associated metadata in this dataset are published in GISAID’s EpiCoV database. To view the contributors of each individual sequence with details such as accession number, Virus name, Collection date, Originating Lab and Submitting Lab and the list of Authors, visit 10.55876/gis8.230214oa. Supplementary digital contents of metadata and fasta sequences are provided as SDC1 and SDC2. EPI_SET_230214oa is composed of 760 individual genome sequences. The collection dates range from 2020-03-06 to 2022-02-02; Data were collected in 1 country and territory; all sequences in this dataset are compared relative to hCoV-19/Wuhan/WIV04/2019 (WIV04), the official reference sequence employed by GISAID (EPI_ISL_402124). Learn more at https://gisaid.org/WIV04.

## References

[1] COVID Live—Coronavirus Statistics—Worldometer. https://www.worldometers.info/coronavirus/.

[2] Global Framework for Urban Water, Sanitation and Hygiene-French.pdf.

[3] Tessema SK (2020). Accelerating genomics-based surveillance for COVID-19 response in Africa. Lancet Microb..

[4] Yang W, Shaman JL (2022). COVID-19 pandemic dynamics in South Africa and epidemiological characteristics of three variants of concern (Beta, Delta, and Omicron). Elife.

[5] Padane, A. *et al.* The dynamics of the COVID-19 pandemic have been driven by several epidemiological waves, determined by the emergence of new SARS-CoV-2 variants of the original viral lineage from Wuhan, China. 69i57.2546j0j15&sourceid=chrome&ie=UTF-8 (2022).

[6] World Health Organisation. Updated working definitions and primary actions for SARSCoV2 variants. Technical document (2023).

[7] Hoteit R, Yassine HM (2022). Biological properties of SARS-CoV-2 variants: Epidemiological impact and clinical consequences. Vaccines..

[8] Mendiola-Pastrana IR, López-Ortiz E, de la Loza-Zamora JGR, González J, Gómez-García A, López-Ortiz G (2022). SARS-CoV-2 variants and clinical outcomes: A systematic review. Life.

[9] Yang W, Shaman JL (2022). COVID-19 pandemic dynamics in South Africa and epidemiological characteristics of three variants of concern (Beta, Delta, and Omicron). ELife..

[10] Abdelnabi R, Boudewijns R, Foo CS, Seldeslachts L, Sanchez-Felipe L, Zhang X, Delang L, Maes P, Kaptein SJF, Weynand B, Vande Velde G, Neyts J, Dallmeier K (2021). Comparing infectivity and virulence of emerging SARS-CoV-2 variants in Syrian hamsters. EBioMedicine..

[11] Khandia R, Singhal S, Alqahtani T, Kamal MA, El-Shall NA, Nainu F, Desingu PA, Dhama K (2022). Emergence of SARS-CoV-2 Omicron (B.1.1.529) variant, salient features, high global health concerns and strategies to counter it amid ongoing COVID-19 pandemic. Environ Res..

[12] Cameroon confirms first coronavirus case. https://www.aa.com.tr/en/africa/cameroon-confirms-first-coronavirus-case/1756866.

[13] Chen Z (2022). Global landscape of SARS-CoV-2 genomic surveillance and data sharing. Nat. Genet..

[14] Njouom R (2021). Coding-complete genome sequence and phylogenetic relatedness of a SARS-CoV-2 strain detected in March 2020 in Cameroon. Microbiol. Resour. Announc..

[15] GISAID: Initiative. https://www.gisaid.org/.

[16] Stoddard G (2022). Using genomic epidemiology of SARS-CoV-2 to support contact tracing and public health surveillance in rural Humboldt County, California. BMC Public Health.

[17] COVID-19 Genomic Surveillance Regional Network: PAHO/WHO | Pan American Health Organization. https://www.paho.org/en/topics/influenza-and-other-respiratory-viruses/covid-19-genomic-surveillance-regional-network.

[18] Cheng X (2022). Identification of SARS-CoV-2 variants and their clinical significance in Hefei. China. Front. Med..

[19] Adepoju P (2021). Challenges of SARS-CoV-2 genomic surveillance in Africa. Lancet Microb..

[20] Robishaw JD (2021). Genomic surveillance to combat COVID-19: Challenges and opportunities. Lancet Microb..

[21] Becker SJ, Taylor J, Sharfstein JM (2021). Identifying and tracking SARS-CoV-2 variants: A challenge and an opportunity. N. Engl. J. Med..

[22] Brito AF (2021). Global disparities in SARS-CoV-2 genomic surveillance. MedRxiv.

[23] Leite JA (2022). Implementation of a COVID-19 Genomic Surveillance Regional Network for Latin America and Caribbean region. PLOS ONE.

[24] Judson FN (1981). Epidemiology of sexually transmitted hepatitis B infections in heterosexuals: A review. Sex. Transm. Dis..

[25] Mbarga NF (2021). Clinical profile and factors associated with COVID-19 in Yaounde, Cameroon: A prospective cohort study. PLOS ONE.

[26] Bwire G (2022). The COVID-19 pandemic in the African continent. BMC Med..

[27] Kopel J (2020). Racial and gender-based differences in COVID-19. Front. Public Health.

[28] Peckham H (2020). Male sex identified by global COVID-19 meta-analysis as a risk factor for death and ITU admission. Nat. Commun..

[29] Mekolo D (2021). Clinical and epidemiological characteristics and outcomes of patients hospitalized for COVID-19 in Douala, Cameroon. Pan Afr. Med. J..

[30] Zaman FA (2021). Exploratory study on the operational issues faced in collection, transportation, and laboratory testing related to COVID-19 in remote areas of selected EAG states of North East and East India. J. Fam. Med. Prim. Care.

[31] Dong R, Hu T, Zhang Y, Li Y, Zhou X-H (2022). Assessing the transmissibility of the new SARS-CoV-2 variants: From delta to omicron. Vaccines.

[32] Chan WS (2022). Geographical prevalence of SARS-CoV-2 variants, August 2020 to July 2021. Sci. Rep..

[33] Tracking SARS-CoV-2 variants. https://www.who.int/activities/tracking-SARS-CoV-2-variants.

[34] Zhan Y, Yin H, Yin J-Y (2022). B.1.617.2 (Delta) variant of SARS-CoV-2: Features, transmission and potential strategies. Int. J. Biol. Sci..

[35] Wang Y (2022). Structural basis for SARS-CoV-2 Delta variant recognition of ACE2 receptor and broadly neutralizing antibodies. Nat. Commun..

[36] Jackson CB, Farzan M, Chen B, Choe H (2022). Mechanisms of SARS-CoV-2 entry into cells. Nat. Rev. Mol. Cell Biol..

[37] Tessema GA (2021). The COVID-19 pandemic and healthcare systems in Africa: A scoping review of preparedness, impact and response. BMJ Glob. Health.

[38] Kwaghe AV (2021). Stigmatization, psychological and emotional trauma among frontline health care workers treated for COVID-19 in Lagos State, Nigeria: A qualitative study. BMC Health Serv. Res..

[39] Freitas-Jesus JV (2022). Stigma, guilt and motherhood: Experiences of pregnant women with COVID-19 in Brazil. Women Birth.

[40] Bhanot D, Singh T, Verma SK, Sharad S (2021). Stigma and discrimination during COVID-19 pandemic. Front. Public Health.

[41] Lin L, Liu Y, Tang X, He D (2021). The disease severity and clinical outcomes of the SARS-CoV-2 variants of concern. Front. Public Health.

[42] Duong D (2021). Alpha, beta, delta, gamma: What’s important to know about SARS-CoV-2 variants of concern?. CMAJ Can. Med. Assoc. J..

[43] Dance A (2022). Omicron’s lasting mysteries: Four questions scientists are racing to answer. Nature.

[44] Wolter N (2022). Early assessment of the clinical severity of the SARS-CoV-2 omicron variant in South Africa: A data linkage study. Lancet.

[45] Mustapha JO (2021). Understanding the implications of SARS-CoV-2 re-infections on immune response milieu, laboratory tests and control measures against COVID-19. Heliyon.

